# Ultrasound-guided nerve blocks in the emergency department

**DOI:** 10.4103/0974-2700.58655

**Published:** 2010

**Authors:** Sanjeev Bhoi, Amit Chandra, Sagar Galwankar

**Affiliations:** Department of Emergency Medicine, Jai Prakash Narayan Apex Trauma Centre, All India Institute of Medical Sciences, New Delhi, India; 1 Department of Emergency Medicine, New York Hospital Queens, Flushing, New York, USA; 2 Faculty of Global Health, University of South Florida, Tampa, Florida, USA

**Keywords:** Ultrasound, nerve block, brachial plexus block, axillary block, forearm block, sciatic block, femoral block, pain control, India

## Abstract

Peripheral nerve blocks preclude the need for procedural sedation and provide adequate anesthesia during painful procedures. This technique can be performed in the emergency department with the aid of ultrasound imaging to identify target nerves. We describe eight cases of upper and lower extremity nerve blocks performed under ultrasound guidance in the emergency department of the Jai Prakash Narayan Trauma Centre, All India Institute of Medical Sciences, New Delhi. Only two of the patients, both with extensive injuries, required any additional anesthesia during the subsequent procedures and all of them reported significant pain control and muscle relaxation.

## INTRODUCTION

Many procedures commonly performed in the emergency department (ED) require regional anesthesia coupled with procedural sedation to achieve adequate patient comfort and cooperation. These procedures include: joint dislocation reduction, fracture reduction, and wound care. Providing field anesthesia with local injections becomes problematic, however, when dealing with large surface areas requiring a volume of medication that approaches toxic doses (e.g., a large laceration repair). Procedural sedation calls for airway monitoring during the procedure and an observation period afterwards, which takes up the time of the emergency physician and other ED staff. Procedural sedation is also associated with rare but potentially devastating side effects, including airway compromise, hypotension, and allergic reactions. Patients with significant comorbidities (e.g., cardiac disease) and those who have had a recent meal have a higher risk of these side effects.

Providing a peripheral nerve block is a safe alternative that utilizes minimal amounts of local anesthetic and does not require hemodynamic monitoring or prolonged post-procedure observation. Inadequate emergency physician training in identifying external landmarks, lack of specialized equipment (e.g., electronic nerve stimulators, which are often used by anesthesiologists to locate nerves), and physician comfort level with the technique has in the past limited the use of this procedure in the ED. Adverse outcomes associated with nerve blocks include hematoma formation, pneumothorax, and localized infection. Ultrasound technology has the potential to address these limiting factors and minimize side effects by allowing for the dynamic visualization of target nerves, needle tip, and the anesthetic as it is infused.

## BACKGROUND

Ultrasound-guided nerve blocks were first described in anesthesiology literature in 1978, when La Grange *et al*. utilized a Doppler device while performing supraclavicular brachial plexus blocks.[1] As ultrasound technology matured over the following two decades, researchers in anesthesiology refocused their attention on this technique. It was not until 1994, however, that sonographic imaging was used to visualize the precise placement of anesthetic into the area surrounding a nerve.[2] Kapral *et al*. performed ultrasound-guided brachial plexus blocks on 40 patients scheduled for hand or wrist surgery and compared the supraclavicular approach with the axillary approach. They achieved 95% surgical anesthesia in both groups, without any reported adverse reactions. Subsequent studies have combined ultrasound imaging with more traditional nerve stimulation devices to confirm placement.[3,4] Chan *et al*. used this combined technique to successfully block the brachial plexus using a supraclavicular approach in 39 of 40 enrolled patients.[4]

In recent years, as the scope of emergency ultrasonography has grown and expertise has increased, many authors have described ultrasound-guided nerve blocks performed in the ED, done without the aid of nerve stimulator devices. In 2006, Blaivas and Lyon described four cases of successful shoulder anesthesia and dislocation reduction performed following ultrasound-guided interscalene brachial plexus blocks.[5] Liebmann *et al*., in the same year, performed ultrasound-guided forearm nerve blocks (radial, median, and ulnar) on 11 patients, none of whom required rescue anesthesia during the subsequent procedures.[6]

Subsequent research further demonstrated both the effectiveness and efficiency of this procedure. In 2008, Stone *et al*. conducted a study on 12 patients with upper extremity emergencies, comparing ultrasound-guided supraclavicular brachial plexus blocks with procedural sedation. Both groups reported adequate anesthesia during their procedures; however, ED length of stay was significantly reduced in the nerve block group: 106 min (95% CI: 57 to 155 min) *vs* 285 min (95% CI: 228 to 343 min).[7]

## METHODS

Following a training seminar on ultrasound-guided upper and lower extremity peripheral nerve blockade, we performed the procedure on eight patients (described below) to assess the feasibility of this technique in our setting. Patients were included in the series over a 3-week period in January 2009. Participants included an emergency medicine faculty physician, senior orthopedics residents, senior internal medicine residents, and senior surgery residents assigned to the ED of the Jai Prakash Narayan Apex (JPNA) Trauma Centre, All India Institute of Medical Sciences (AIIMS), New Delhi. The trauma center ED has its own dedicated SonoSite MicroMaxx® ultrasound machine with a variety of interchangeable probes. Sterile technique was strictly adhered to and, unless otherwise indicated, the high-frequency linear probe was utilized during the procedures.

## CASE 1

A 31-year-old male with a history of four right shoulder dislocations in the past following an injury sustained as a child, presented with yet another right shoulder dislocation. A right brachial plexus nerve block was performed under ultrasound guidance, using a supraclavicular approach. The first attempt using an in-plane approach (with the needle parallel to the plane of the ultrasound image) was aborted when the needle could not be visualized within the image. An out-of-plane (needle perpendicular to the image) approach was then attempted and 15 ml of 2% xylocaine was injected around the nerve. The patient reported paresthesias down his arm during the injection and then an immediate reduction in pain in his right shoulder by about 85%. His shoulder was then easily reduced using the traction–countertraction technique. He reported significantly better anesthesia during the procedure than during his previous shoulder reductions. He was discharged from the ED after a 1-h observation period and after he reported full return of sensation to his right upper extremity.

## CASE 2

**Figure 1:**
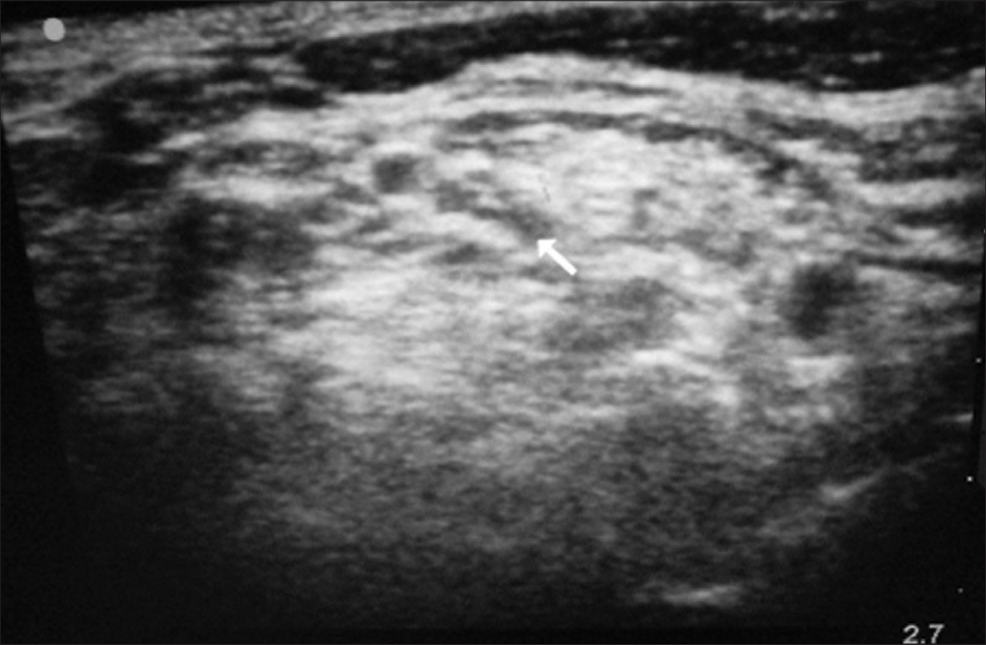
Brachial plexus nerve block, anterior scalene approach

An 82-year-old female presented to the ED with a right anterior shoulder dislocation following a fall down a flight of stairs. A right brachial plexus block was performed under ultrasound guidance using the anterior scalene approach [Figure 1]. Using the out-of-plane approach, 8 ml of 2% xylocaine was infused around the nerve bundle. The patient reported complete right arm anesthesia and her shoulder was reduced easily using the external rotation and abduction approach. She was kept for observation for 1 h and discharged following return of sensory function.

## CASE 3

**Figure 2:**
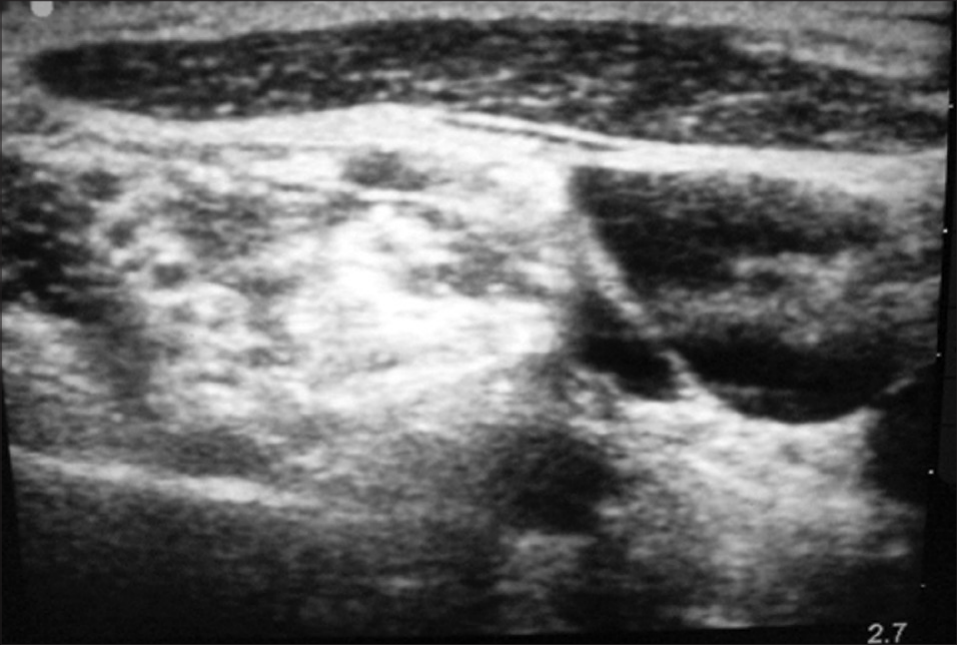
Brachial plexus nerve block, anterior scalene approach

A 40-year-old male presented to the ED with an anterior shoulder dislocation following a fall down two steps. A right brachial plexus block was performed under ultrasound guidance using the anterior scalene approach [Figure 2]. In Figure 2, the brachial plexus is seen located within the anterior scalene muscle, deep to the sternocleidomastoid muscle (the most superficial structure in this image) and lateral to the internal jugular vein. Using the out-of-plane approach, 8 ml of 2% xylocaine was placed around the nerve bundle. The dislocated shoulder was reduced using the external rotation and abduction method. The patient reported zero pain during reduction. He was discharged after 1 h of observation and return of sensory function.

## CASE 4

**Figure 3:**
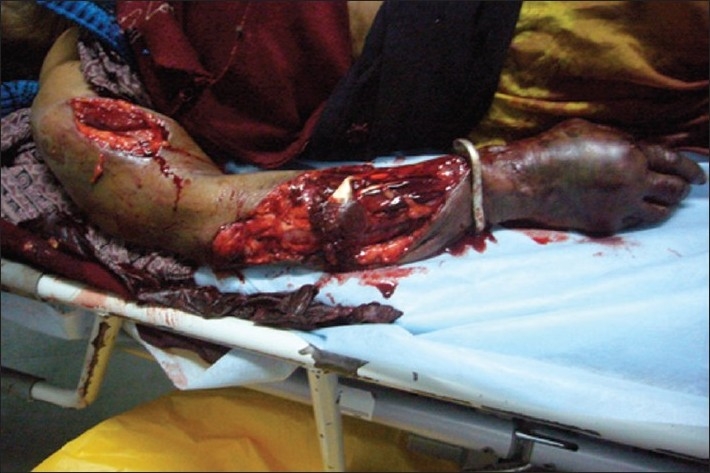
Crush injury to right arm

**Figure 4:**
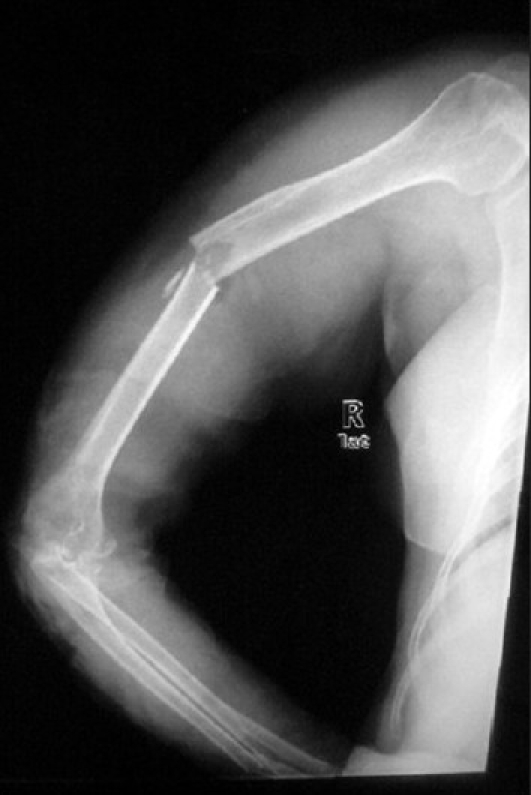
Crush injury to right arm

**Figure 5:**
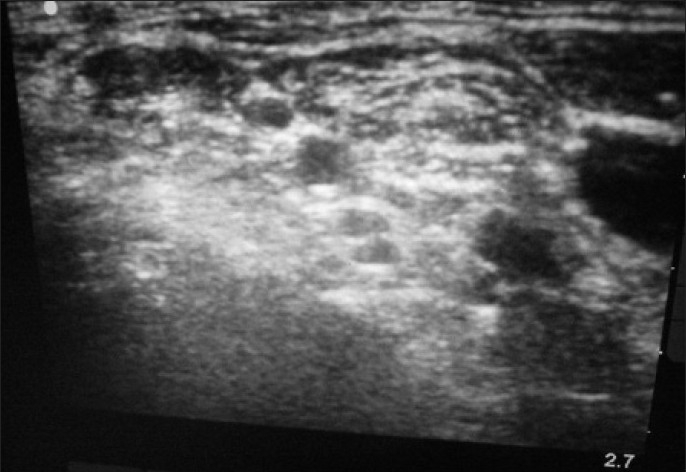
Brachial plexus nerve block, anterior scalene approach

A 65-year-old female presented to the ED after having been struck by a bus while walking on the road. She had a crush injury with open fractures of her right upper arm and forearm. A steel bracelet was noted to be impacted in the macerated tissue around her wrist [Figures 3 and 4]. She was in severe pain, screaming for help upon arrival. A right brachial plexus block using the anterior scalene approach was performed under ultrasound guidance [Figure 5]. The brachial plexus is identifiable in the center of this image, within the anterior scalene muscle and lateral to the internal jugular vein, forming a butterfly shape. An out-of-plane approach was used to infuse 6 ml of 2% xylocaine. Following this, the patient reported complete right upper extremity anesthesia for approximately 45 min. During that time, an orthopedics senior resident was able to manipulate her arm, extract the bracelet from her wrist, irrigate the wound, and perform primary closure of the wound and splinting (as a temporizing treatment prior to operative management).

## CASE 5

**Figure 6:**
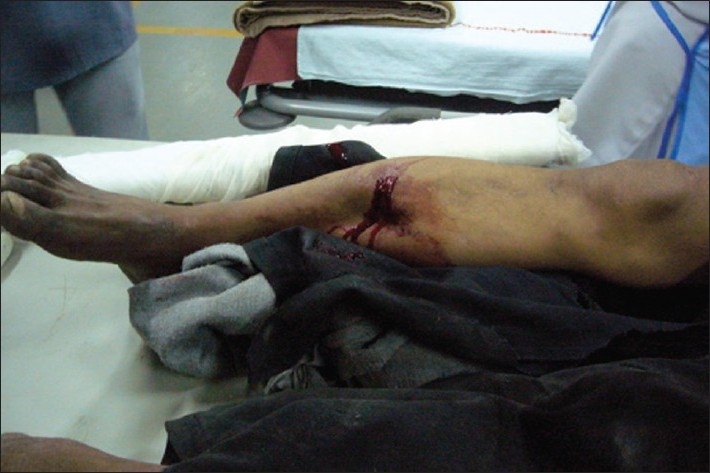
Open tibia-fibula fracture

**Figure 7:**
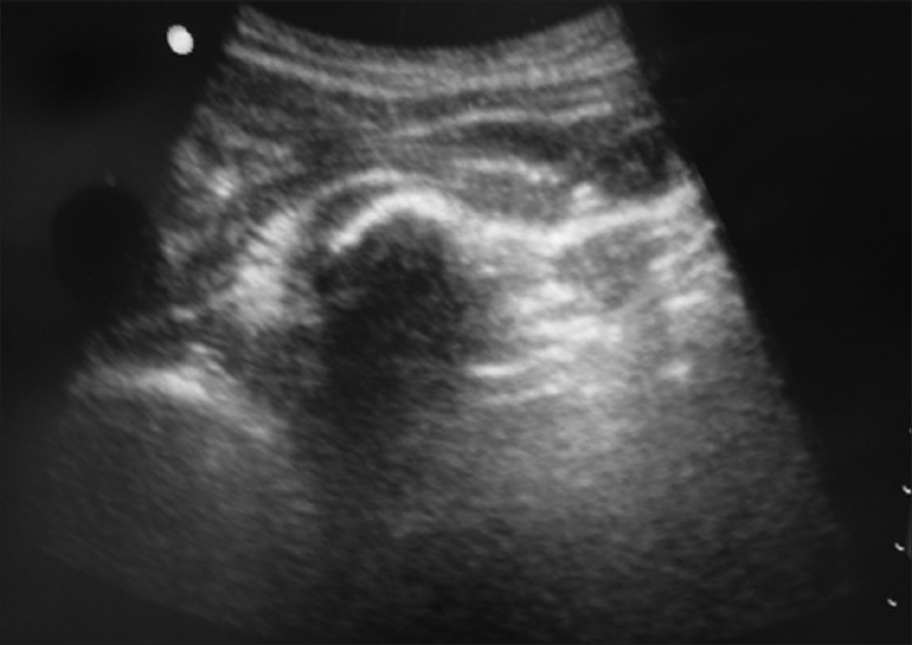
Sciatic nerve block

**Figure 8:**
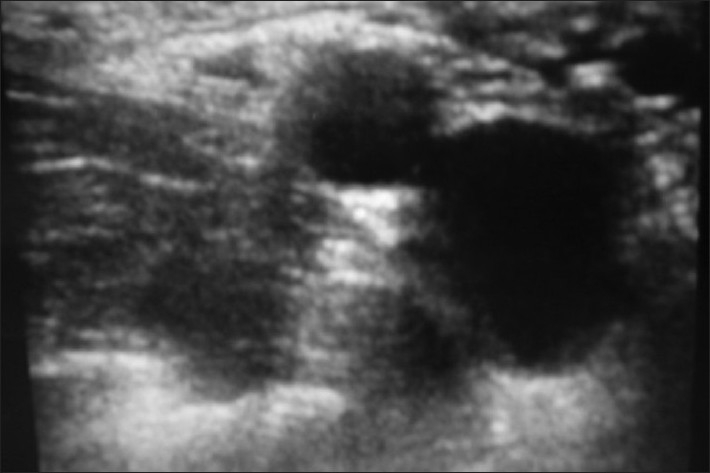
Femoral nerve block, femoral nerve, artery, and vein

A 35-year-old male presented in the ED with an open fracture of the right tibia and fibula following an assault with a field hockey stick [Figure 6]. The patient was first placed in a temporary splint to control pain with leg positioning. A sciatic nerve block was performed under ultrasound guidance [Figure 7]. The sciatic nerve in this image is located deep to the gluteus maximus muscle and is seen between the ischial tuberosity and the greater trochanter. Using an out-of-plane approach and a curved array abdominal probe, 8 ml of 2% xylocaine was infused around the sciatic nerve. The patient reported only mild to moderate pain during reduction. A femoral nerve block was then performed under ultrasound guidance [Figure 8]. The nerve here is the triangular structure, lateral and anterior to the femoral artery and vein. Using the linear high-frequency probe and an in-plane approach, 5 ml of 2% xylocaine was infused around the femoral nerve. Following this, the patient reported further alleviation of his pain. He was then transferred to the x-ray suite, his wound was irrigated, and his leg was placed in a formal splint, pending further operative treatment.

## CASE 6

**Figure 9:**
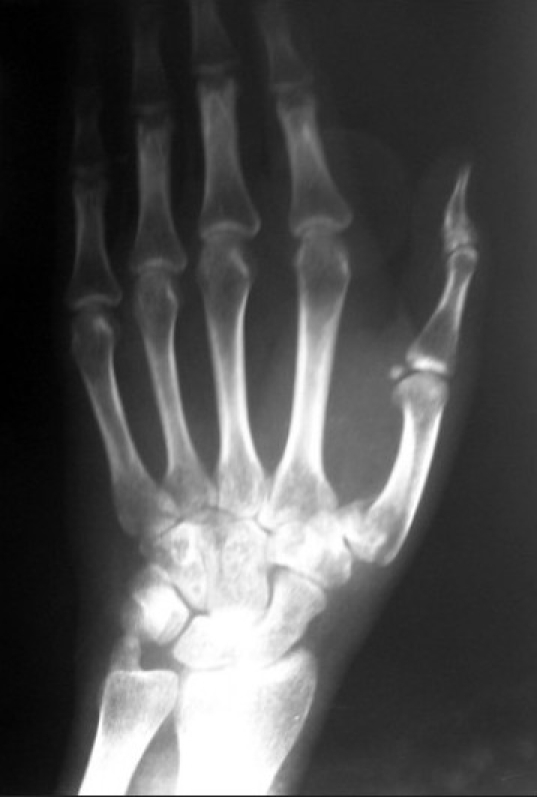
Bennett's fracture

**Figure 10:**
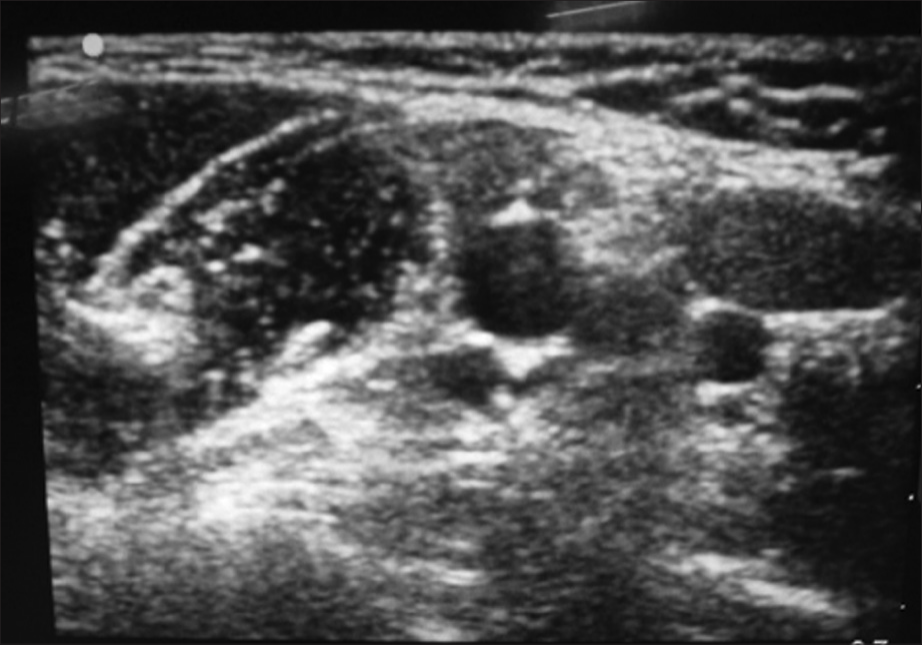
Axilliary nerve block, musculocutaneous, median, ulnar, and radial nerves

A 33-year-old male with a history of renal transplant presented in the ED with a Bennett fracture of the left hand [Figure 9]. The injury had been sustained following a fall 10 days prior to arrival. A forearm approach for the nerve block was not attempted since the patient had an arteriovenous (A-V)fistula at this site. An axillary nerve block was performed under ultrasound guidance [Figure 10]. Using the in-plane approach, a total of 10 ml of 2% xylocaine was infused into the space surrounding the musculocutaneous, medial, radial, and ulnar nerves. The patient reported complete cutaneous anesthesia in the affected limb, with zero sensation to pinprick. However, a prolonged and aggressive reduction was required, given the duration of injury, and the patient reported moderate pain during this process. The patient was discharged following return of sensation and a 1-hour observation period.

## CASE 7

**Figure 11:**
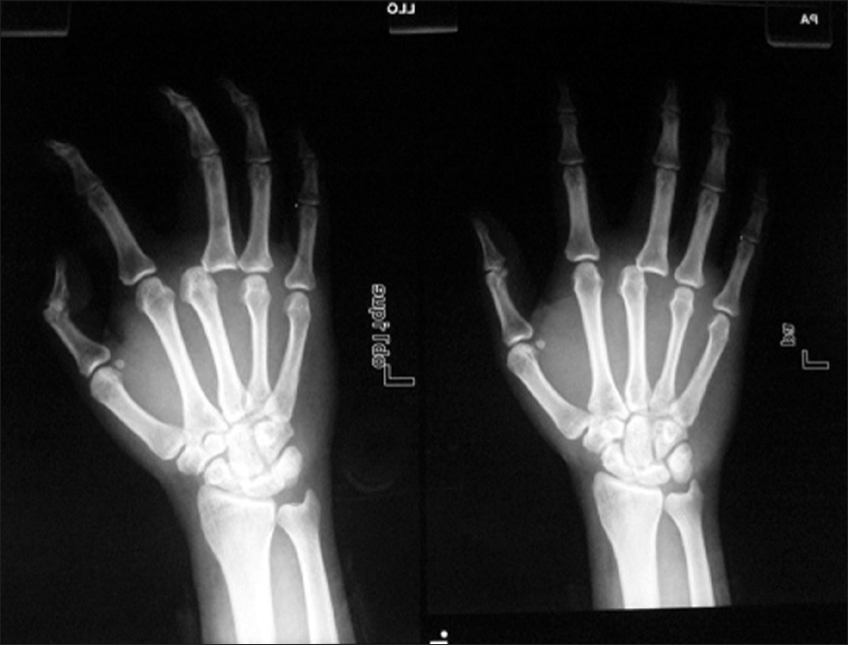
Proximal phalanx dislocation

**Figure 12:**
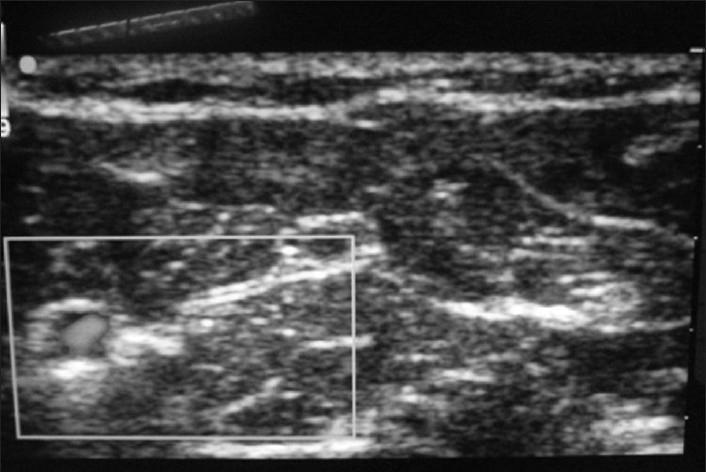
Forearm nerve block, radial artery and median nerve

**Figure 13:**
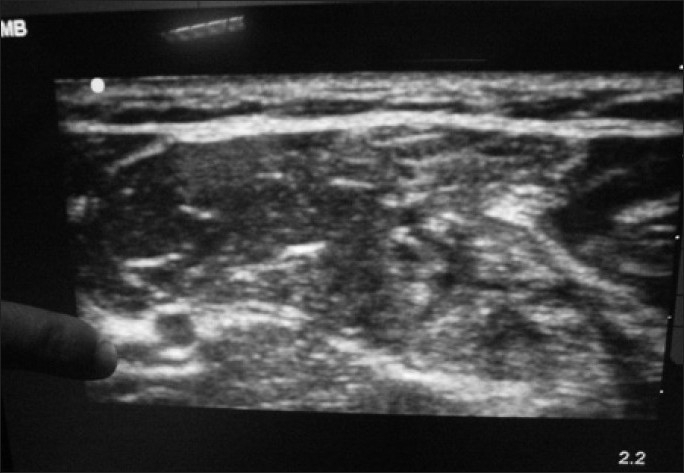
Forearm nerve block, ulnar artery and ulnar nerve

A 36-year-old male presented with a proximal phalanx dislocation of the left third digit following a fall during a seizure 3 days prior to presentation [Figure 11]. A left forearm nerve block of median and ulnar nerves was performed under ultrasound guidance [Figures 12 and 13]. In Figure 12, the radial artery is identified with color Doppler; the median nerve is located in the center of the image. In Figure 13, the ulnar nerve is located adjacent to the ulnar artery to the left of the image. The median nerve block was performed with 3 ml of 2% xylocaine using the out-of-plane approach. The ulnar nerve block was performed with 3 ml of 2% xylocaine using the in-plane approach. The patient reported hand paresthesias during the injection process and then noted an immediate loss of sensation throughout the affected area. Reduction of the phalanx was performed following the block. Prolonged and aggressive traction was required, which was expected, given the period of time since the initial injury. The patient reported zero pain associated with the reduction process. The patient was reevaluated 30 min after the procedure and had complete return of sensation and function of his left hand.

## CASE 8

**Figure 14:**
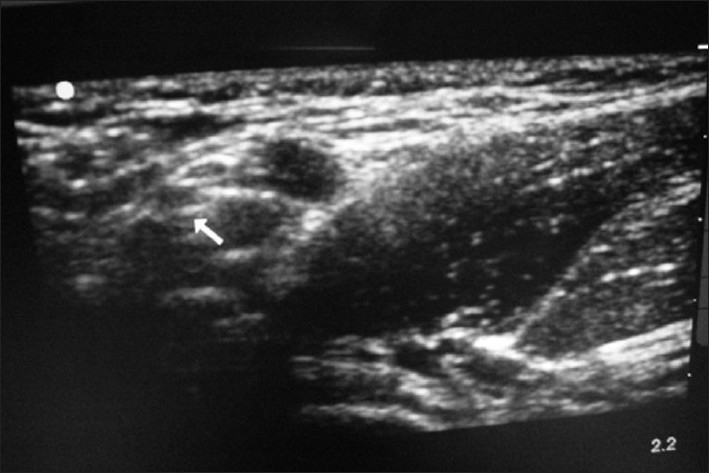
Axilliary nerve block, ulnar, radial, and median nerves

**Figure 15:**
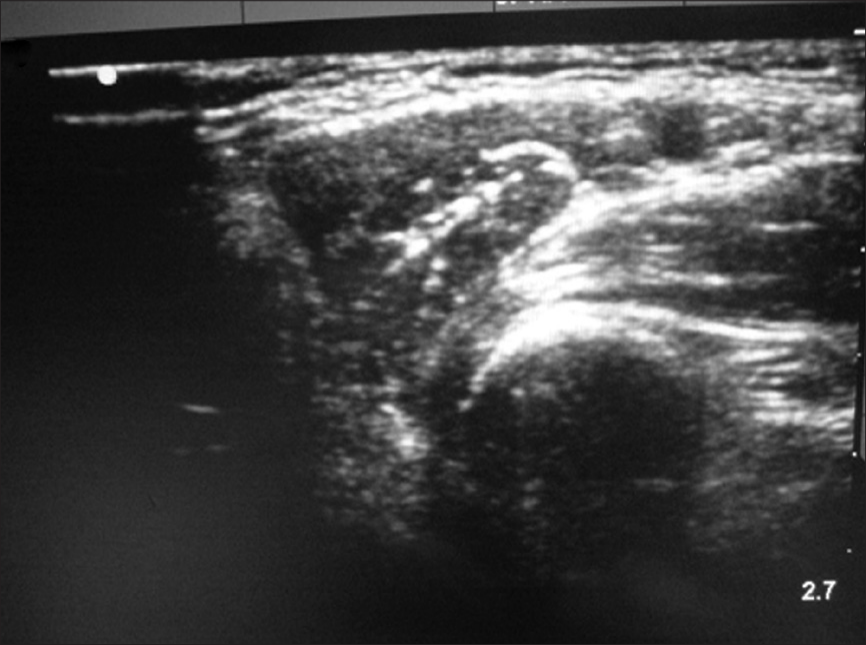
Axilliary nerve block, musculocutaneous nerve

**Figure 16:**
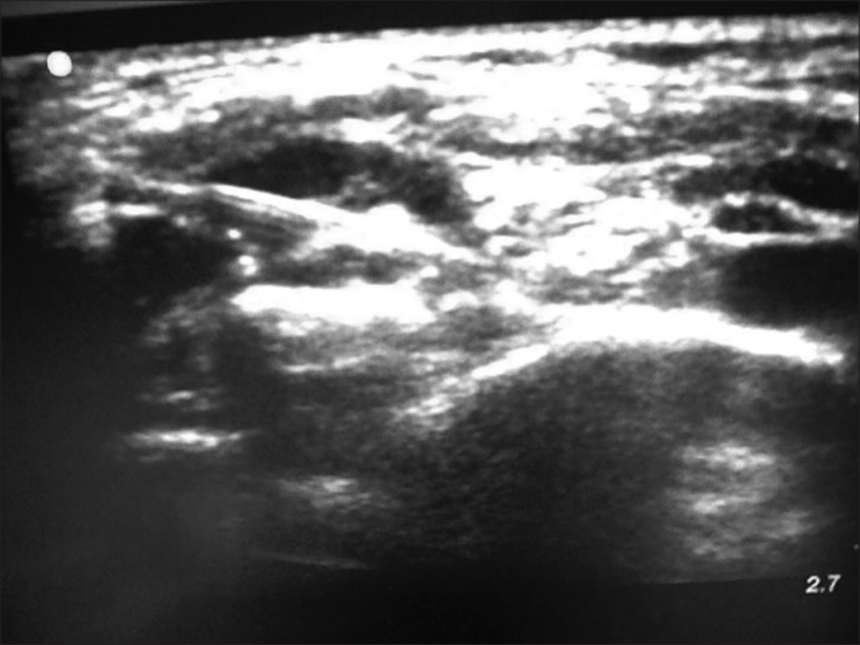
Brachial plexus nerve block, supraclavicular approach

A 10-year-old male presented to the ED for a follow-up evaluation following a right forearm mid-shaft radius and ulnar fracture 6 days prior to presentation. A follow-up x-ray demonstrated worsened displacement, requiring reduction in the ED. An axillary nerve block was performed under ultrasound guidance [Figures 14 and 15]. In Figure 14, the ulnar and radial nerves can be seen located posterior to the brachial artery, with the median nerve located anterior to it. In Figure 15, the musculocutaneous nerve is the hyperechoic structure located in the center of the coracobrachialis muscle. Using an in-plane approach, 3 ml of 2% xylocaine was placed around the ulnar and radial nerves. Due to poor patient cooperation at this point, the median nerve could not be approached. Incomplete right upper extremity anesthesia was noted after this initial block and so a supraclavicular brachial plexus block was performed under ultrasound guidance [Figure 16] using an in-plane approach. This resulted in complete sensory blockade of the right upper extremity. In Figure 16, the needle can be visualized in the image delivering the anesthetic to the nerve bundle lateral to the vessels. The patient's splint was removed and the fracture area was manipulated with no reported pain by the patient. The patient did however report pain during the subsequent reduction process. Full return of sensation and distal motor function was noted when patient was re-evaluated 30 min after the block.

## DISCUSSION

Ultrasound-guided nerve blocks are a new concept for EDs, and a relatively new one for anesthesiologists. This technique was selected for these patients to demonstrate its efficacy in terms of pain management and efficiency in terms of utilization of resources. In particular, these patients benefited from the avoidance of procedural sedation, reducing their length of stay and precluding the need for intravenous access and airway monitoring. In patients with severe injuries (e.g., the patients with the right arm crush injury and the open tibia/fibula fracture), small doses of intravenous pain control agents were given to supplement the analgesia provided by the nerve block, but our study participants felt that the need for these additional medications was minimized by the use of the blocks.

Cases 1, 2, and 3 benefited in particular since they presented with simple shoulder dislocations. No intravenous access or supplemental analgesia was necessary for these patients. All of the subsequent reductions were easily completed with minimal patient discomfort. These patients were not exposed to the risks and prolonged observation associated with procedural sedation.

The patients with severe injuries in our series (Cases 4 and 5) also benefited from the regional anesthesia provided by the nerve block. These patients presented with conditions requiring surgical intervention, but their injuries required a significant amount of manipulation in the ED. The nerve blocks in these situations were the ideal adjunct to intravenous therapies, allowing wound irrigation and closure and fracture splinting, once again without the need for procedural sedation.

The patients described in Cases 6 (Bennett fracture) and 7 (phalanx dislocation) presented with complicated injuries. They both presented several days after their initial injuries and so a prolonged reduction was anticipated. Without some sort of muscle relaxation and analgesia, these reductions would likely have been unsuccessful. Considering procedural sedation for such localized injuries, however, would have exposed the patients to unnecessary risks. Regional anesthesia via ultrasound-guided nerve blocks provided adequate pain control and allowed successful reduction.

Despite our successes with the patients described previously, our experience with the patient in Case 8 was mixed. As the patient was a child, it was difficult to persuade him to cooperate with the axillary nerve block and the procedure could not be completed. When he calmed down, a supraclavicular block was performed to complete the anesthesia. Despite the full cutaneous anesthesia that followed, he reported pain during the subsequent forearm reduction. His high level of anxiety during the regional block and the reduction process itself made it difficult to gauge how much he benefited from the procedure.

Our Case Series signals the opportunity of broadening the scope of using the ultrasound in the ED. The use of dynamic sonographic imaging during the nerve block process allowed for the precise application of limited amounts of anesthetic agents. Study participants utilizing this method after minimal training felt confident of their ability to identify surrounding structures and of their injection technique.

## CONCLUSION

Our case series demonstrated the feasibility of this technique in Indian EDs that are equipped with bedside ultrasound machines. Residents with minimal training and experience in ultrasonography were able to successfully perform this procedure to achieve adequate regional anesthesia. Further trials are required to evaluate the efficacy and safety profile of this procedure when performed in the ED and to compare the efficacy with alternative techniques.

The success of this case series demonstrates the importance of ultrasound training in the curriculum of emergency medicine as it develops as a specialty in India. Emergency ultrasonography training should include core areas (e.g., the FAST exam - Focused Assessment with Sonography for Trauma) and an expanded set of bedside applications (e.g., vascular access, nerve blocks, etc.). This process must start in academic emergency medicine departments and then be disseminated to smaller hospitals. This progression will be enhanced as ultrasound technology advances and becomes more cost-effective.
